# Strategic placement of volunteer responder system defibrillators

**DOI:** 10.1007/s10729-024-09685-4

**Published:** 2024-09-10

**Authors:** Robin Buter, Arthur Nazarian, Hendrik Koffijberg, Erwin W. Hans, Remy Stieglis, Rudolph W. Koster, Derya Demirtas

**Affiliations:** 1 Center for Healthcare Operations Improvement and Research, University of Twente, Enschede, The Netherlands; 2 Industrial Engineering and Business Information Systems, University of Twente, Enschede, The Netherlands; 3 Health Technology & Services Research, University of Twente, Enschede, The Netherlands; 4 Department of Cardiology, Amsterdam UMC Location University of Amsterdam, Amsterdam, The Netherlands; 5 Helmond, The Netherlands

**Keywords:** Facility location, Emergency, Partial cover, Volunteer responder system, Automated external defibrillator, Out-of-hospital cardiac arrest, Operations research, Operations management, Optimization

## Abstract

Volunteer responder systems (VRS) alert and guide nearby lay rescuers towards the location of an emergency. An application of such a system is to out-of-hospital cardiac arrests, where early cardiopulmonary resuscitation (CPR) and defibrillation with an automated external defibrillator (AED) are crucial for improving survival rates. However, many AEDs remain underutilized due to poor location choices, while other areas lack adequate AED coverage. In this paper, we present a comprehensive data-driven algorithmic approach to optimize deployment of (additional) public-access AEDs to be used in a VRS. Alongside a binary integer programming (BIP) formulation, we consider two heuristic methods, namely Greedy and Greedy Randomized Adaptive Search Procedure (GRASP), to solve the gradual Maximal Covering Location (MCLP) problem with partial coverage for AED deployment. We develop realistic gradually decreasing coverage functions for volunteers going on foot, by bike, or by car. A spatial probability distribution of cardiac arrest is estimated using kernel density estimation to be used as input for the models and to evaluate the solutions. We apply our approach to 29 real-world instances (municipalities) in the Netherlands. We show that GRASP can obtain near-optimal solutions for large problem instances in significantly less time than the exact method. The results indicate that relocating existing AEDs improves the weighted average coverage from 36% to 49% across all municipalities, with relative improvements ranging from 1% to 175%. For most municipalities, strategically placing 5 to 10 additional AEDs can already provide substantial improvements.

## Highlights

- Unlocks the full potential of Volunteer Response Systems for cardiac arrests by finding (near-)optimal locations of Automated External Defibrillators (AEDs) to be retrieved by dispatched volunteers using different modes of transportation.
- Formulates and applies continuous coverage decay functions for each mode of transportation, instead of the commonly used binary coverage for cardiac arrests.
- Applies a data-driven algorithmic approach to 29 real-world instances (municipalities) from the Netherlands, including cardiac arrests in both public and residential locations.
- Informs policymakers of the benefits of deploying additional AEDs and identifies areas that currently lack coverage.

## Introduction

Cardiovascular disease is one of the leading causes of premature death in the world [1]. Cardiac arrest occurs when the heart is suddenly unable to effectively pump blood throughout the body due to loss of heart function. Each year in the United States, more than 350,000 people have an out-of-hospital cardiac arrest (OHCA), assessed by emergency medical services (EMS) [2]. In Europe, between 350,000 and 700,000 OHCAs are reported per year [3]. A recent meta-analysis shows that only 10.7% of all OHCA patients in Europe survive to hospital discharge [4]. Given its prevalence and low survival rates, OHCA is recognized as an important public health problem [5, 6].

Although overall survival rates are low, early cardiopulmonary resuscitation (CPR) and early defibrillation using an automated external defibrillator (AED) drastically increase survival-to-discharge rates [3, 7, 8]. It is estimated that each minute of delay in defibrillation decreases the probability of survival by roughly 10% [9]. Since EMS often cannot arrive timely in case of a cardiac arrest, bystanders play a crucial role in enhancing survival by initiating treatment, performing CPR and delivering a shock using an AED [7, 10]. However, a nearby (on-site) AED is often not available or accessible to bystanders [11–13]. Sondergaard et al. [14] show that the probability of bystander defibrillation in public areas decrease steeply after 100 meter distance to the AED. In residential areas, where more than 70% of the cardiac arrests occur [14, 15], bystanders defibrillated a patient in merely 1.2% of the cases in Denmark [14].

To increase the chance of early defibrillation, volunteer responder systems (VRS) have been introduced in several countries. In the Netherlands, an alert system called HartslagNu was developed to decrease the time to defibrillation, particularly in residential areas [16, 17]. A dispatch center activates this system when there is a reasonable suspicion that the emergency call is related to a cardiac arrest. Registered volunteer responders^1^ that are within a 2000 meter radius of the cardiac arrest receive a text message or an alert via the smartphone application. If a dispatched volunteer is near a registered AED, the volunteer also receives the location of the AED and is asked to retrieve it. Based on the surveys completed by volunteers in the Netherlands, it is known that volunteer responders may retrieve the AED on foot, by bike, or by car. Stieglis et al. [15] show that the median time to shock decreases from 10:59 to 8:17 min for cardiac arrest cases in which at least one volunteer was assigned an AED.

Having an AED nearby is crucial for enhancing the effectiveness of public access defibrillation (PAD) programs. Nonetheless, the cost of purchasing and maintaining AEDs makes it impractical to deploy them everywhere. Ringh et al. [18] and Demirtas et al. [19] argue that AED coverage should prioritize high risk areas for cardiac arrests to successfully implement a PAD program. Therefore, optimal positioning of AEDs is essential to unlock the full potential of a VRS.

In this paper, we propose a comprehensive data-driven algorithmic approach to optimize the deployment of public-access AEDs to be used in a VRS. Although recent literature on optimization methods for guiding AED deployment has shown the potential of mathematical optimization [20–23], no previous research has addressed dispatched volunteers in a VRS. In particular, we develop a realistic coverage function that can be used in facility location models, accounting for transportation mode and distance decay. Additionally, an estimation of how much time a volunteer has on average to retrieve an AED before EMS arrives is provided. Based on this we formulate a gradually decreasing coverage function for each mode of transportation to model the decreasing effectiveness of AEDs as distance to the victim increases. To obtain a spatial distribution of cardiac arrest incidence, we apply kernel density estimation (KDE) to historical cardiac arrest data in both public and residential areas. From that distribution both training and evaluation sets of cardiac arrests are sampled. We create candidate AED locations in a uniform grid across the study region. It is known that temporal (in)accessibility of AEDs is an issue [24], so we assume that these AED locations are accessible 24/7 since AEDs can be placed outside in (secured) cabinets. In addition to a binary integer programming (BIP) formulation, two heuristic methods are considered, namely Greedy and Greedy Randomized Adaptive Search Procedure, to solve the gradual Maximal Covering Location Problem for AED deployment. We show, using a large problem instance, that these heuristics can obtain a solution within 0.18% of the BIP formulation’s solution in 88% less time. In addition to that, the heuristics are able to solve larger problem instances than BIP formulation with the same computer memory. This implies that a potentially better solution can be found by increasing the granularity of candidate locations or demand points.

To demonstrate the effectiveness of our approach, we apply it to 29 real-world instances from the Netherlands. By using actual cardiac arrest and AED data, we optimize AED locations in 29 municipalities in the North Holland region. Moreover, we formulate a timeline of events during a cardiac arrest to model a coverage function for volunteers traveling on foot, by bicycle, and by car.

This paper has the following structure. Section 2 reviews the related literature. Section 3 describes the formulation of the Maximal Covering Location Problem with a partial coverage, the exact method and the heuristics. Section 4 introduces the data, explains our approach to formulating the coverage functions, and the procedure to generate cardiac arrest locations. Section 5 then presents the experiment design and provides the numerical results. Finally, Section 6 discusses the finding while Section 7 concludes the paper.

## Related literature

Covering location models are among the most prominent facility location models and have been favored for their applicability in practice, especially for the deployment of emergency facilities [25]. Despite this, Ahmada-Javid et al. [26] concluded that merely 5% of the articles on healthcare facility location focused on public access devices for medical emergencies. For the AED deployment problem, where the number of facilities is limited and each facility has a maximum service distance, the Maximal Covering Location Problem (MCLP) [27] is particularly suitable. In the MCLP, the goal is to find the optimal locations for a set number of facilities to maximize the total demand covered. Demand is considered covered if a facility lies within a specified service distance. Replacing this binary coverage function with either a non-increasing step function or a gradually decaying coverage function was proposed by Church and Roberts [28], Berman and Krass [29], Berman et al. [30], and Karasakal and Karasakal [31]. Other extensions of the MCLP could include probabilistic demand weights [32, 33], chance constraints to address demand uncertainty [34], or expected coverage as a measure of the probability that a facility is available [35]. Instead of focusing on coverage, Erkut et al. [36] used a survival function to maximize the expected number of cardiac arrest survivals when determining locations for emergency medical service stations.

While the medical community has emphasized the importance of available AEDs (e.g. [37]), literature on optimization techniques for guiding AED deployment remains limited. As one of the first to apply mathematical optimization to AED deployment, Mandell and Becker [38] proposed a multi-objective ILP to determine an equitable distribution of AEDs among basic life support units. Rauner and Bajmoczy [39] evaluated the cost effectiveness of placing AEDs in ambulances by developing a decision model in combination with an integer programming model. On-site AED placement was first modeled by Myers and Mohite [40], who applied the MCLP model to a small case study on a university campus. Chan et al. [41] demonstrated that an MCLP approach, using clusters of historic cardiac arrest locations, outperforms a population-guided method in Toronto. Sun et al. [24] extended this model to include the temporal availability of AEDs. Later, Sun et al. [23] applied the models of [24, 41] to Danish data, showing that optimized AED locations significantly improve 30-day survival rates. Chan et al. [20] used an exponential coverage decay function and formulated three different models that consider how bystanders may retrieve AEDs, which concern multiple responders, single-responder worst case, and single-responder best case. Moreover, Chan et al. [21] proposed a row-and-column generation algorithm to determine a robust solution, optimizing AED locations for the worst-case spatial distribution of cardiac arrests. Tierney et al. [42] modeled the cost trade-off between purchasing additional AEDs and relocating existing AEDs.

Exact methods are typically used to solve smaller covering problems, but heuristic approaches may be necessary for larger problem instances. Berman and Krass [29] showed through test cases that a Greedy algorithm often provides optimal or near-optimal solutions for the generalized MCLP. Greedy Randomized Adaptive Search Procedure (GRASP) [43] was used by Resende [44] to solve covering problems. GRASP is a multi-start heuristic that uses local search to iteratively improve initial solutions constructed by randomized greedy. Genetic Algorithms (GA) have also been employed successfully [45–48].

We observe that most studies focus on cardiac arrests occurring in public spaces, and ignore residential areas [20, 21, 23, 40, 41]. However, more than 70% of the cardiac arrest occur in residential areas [14, 15]. Similarly, these studies also used a fixed set of candidate locations, typically public buildings, which may not provide adequate potential for coverage in residential areas. Tierney et al. [22] addressed both issues by including cardiac arrests in residential areas and using candidate locations that included residential buildings. Nowadays, it is common practice to place AEDs outdoors in (secured) cabinets.

Although the definition of the coverage function is a key element in the MCLP, we observe that most studies made simplistic assumptions about the maximal service distance and function’s shape. Most studies used binary coverage with a radius of either 100m [22–24, 41] or 176.25m [49]. Chan et al. [20] considered binary coverage unrealistic and opted for an exponential coverage decay function with a cutoff point at 100m and with its shape mimicking the survival curve of cardiac arrests. The decision to use 100m as a cutoff is not based on data. Additionally, no studies addressed dispatched volunteers in a VRS, focusing solely on bystanders. Surveys from the Netherlands show that dispatched volunteers in a VRS may use various modes of transportation, necessitating multiple coverage functions.

Our contributions are as follows. (1) We propose an elaborate method to formulate a coverage function for the deployment of AEDs, based on a timeline of activities during the activation of a VRS and introducing various modes of transportation beyond walking. (2) We present an exact BIP and two heuristic methods to solve the MCLP with partial coverage for AED deployment. The BIP formulation of the MCLP is difficult to solve for larger instances, resulting in a considerable computation time and memory demands. We demonstrate that the heuristics can obtain a solution that performs within 0.18% of the BIP formulation’s solution in 88% less time for large problem instances. (3) We apply our methodology to cardiac arrests in both public and residential areas, addressing a gap in previous research that often overlooked residential cases, to improve public health.

## Methods

### Maximum coverage location problem with gradual coverage decay

As discussed in Section 2, the AED deployment problem can be interpreted as a Maximal Covering Location Problem (MCLP). This formulation assumes binary coverage, meaning a demand point either receives full coverage from a facility or none at all. Consequently, cardiac arrests close to or far from the AED receive identical coverage if within the coverage radius, while those just outside the range receive no coverage at all. Given that the probability of surviving a cardiac arrest decreases rapidly with time, it is logical that a cardiac arrest closer to an AED should receive a higher coverage than one farther away. Therefore, we model this problem as a gradual coverage decay extension of the MCLP, as first introduced by Berman et al. [29] and Karasakal and Karasakal [31]. We assume that purchasing, installing, and maintenance costs of AEDs are equal and independent of location.

Let $\mathcal {I}$ denote the set of demand points and $\mathcal {J}$ denote the set of facility locations. Let $\mathcal {J}^e$ then denote the locations of existing facilities and $\mathcal {J}^c$ denote the candidate locations for new facilities, such that $\mathcal {J} = \mathcal {J}^e \cup \mathcal {J}^c$ and $\mathcal {J}^e \cap \mathcal {J}^c= \emptyset$. Furthermore, let $d_{ij}$ be the distance between demand *i* and facility *j*. We define a coverage decay function *f* that maps a distance $d_{ij}$ to a coverage value between [0, 1]. We assume that this function is monotonically decreasing with distance and has a cutoff point *r*, meaning that every distance greater than *r* has a coverage value of 0. Also, let $\mathcal {J}_i \subseteq \mathcal {J}$ denote the set of locations *j* that can cover demand *i*, $\mathcal {J}_i = \left\{ j \in \mathcal {J} \mid f(d_{ij}) > 0 \right\}$.

Furthermore, we define *N* as the total number of additional facilities that can be opened. We define binary variables $Y_j$ to be 1 if location $j\in \mathcal {J}$ is opened and 0 otherwise, and $X_{ij}$ to be 1 if demand *i* is covered by facility $j\in \mathcal {J}$ and 0 otherwise. Then, the MCLP with gradual coverage can be formulated as follows:

1a $$\begin{aligned}&\text {Maximize} \sum _{i \in \mathcal {I}}{\sum _{j \in \mathcal {J}_i} f(d_{ij})X_{ij}}&\end{aligned}$$

1b $$\begin{aligned}&\text {Subject to}&\nonumber \\&\sum _{j \in \mathcal {J}^c}{Y_j} \le N&\end{aligned}$$

1c $$\begin{aligned}&Y_j = 1&\forall j \in \mathcal {J}^e \end{aligned}$$

1d $$\begin{aligned}&X_{ij} \le Y_j&\forall i \in \mathcal {I}, j \in \mathcal {J}_i \end{aligned}$$

1e $$\begin{aligned}&\sum _{j \in \mathcal {J}_i}{X_{ij}} \le 1&\forall i \in \mathcal {I} \end{aligned}$$

1f $$\begin{aligned}&X_{ij} \in \{0, 1\}&\forall i \in \mathcal {I}, j \in \mathcal {J}_i \end{aligned}$$

1g $$\begin{aligned}&Y_j \in \{0, 1\}&\forall j \in \mathcal {J} \end{aligned}$$

The objective function (1a) maximizes the total coverage. Constraints (1b) ensure that up to *N* additional facilities are open. Moreover, constraints (1c) force the existing facilities to stay open. Constraints (1d) also model that a demand point can only be covered by a facility that is open. Additionally, constraints (1e) ensure that each demand point can only be covered by at most one facility, meaning that for this maximization problem, it will be the facility that provides the highest coverage. Finally, constraints Eqs. 1f and 1g impose binary restriction on the decision variables.

We extend this formulation to model the different modes of transportation that a volunteer may use. Let *T* denote the set of modes of transportation and let $w_t$ be the fraction of volunteers that would travel by mode $t \in \mathcal {T}$, with $\sum _{t \in \mathcal {T}}{w_t} = 1$. Furthermore, we define a specific coverage function $f^t$ for each of the modes of transportation. So, we redefine the coverage function *f* to Eq. 2.

2 $$\begin{aligned} f(d_{ij}) = \sum _{t \in T}w_tf^t(d_{ij}) \end{aligned}$$

### Heuristic approaches

The formulation described in Section 3.1 does not scale well with larger problem instances. Therefore, this section introduces heuristic approaches that deliver near-optimal solutions with significantly lower computational costs, suitable for larger problem sizes. In Section 3.2.1, we propose a Greedy algorithm for AEDs deployment. In Section 3.2.2 we propose a GRASP algorithm which extends the Greedy algorithm by incorporating randomness during a construction phase and improving the solution through a local search procedure.

#### Greedy algorithm

Let $c_{ij} = f(d_{ij}) = \sum _{t \in T}w_tf^t(d_{ij})$ be the coverage values. Among the open facilities in $\mathcal {J}_i$, which includes existing facilities, the one that provides the highest coverage to demand point *i* is indicated by the variable $J_i^* = \mathop {\mathrm {\arg \!\max }}\limits _{\left( j \in \mathcal {J}_i \mid Y_j = 1\right) } c_{ij}$, for all $i \in \mathcal {I}$. If no facility can cover demand point *i* (i.e. $\mathcal {J}_i = \emptyset$), then $J_i^* = \text {nil}$. Let $\mathcal {I}_j \subseteq \mathcal {I}$ denote the subset of demand points that can be covered by a facility at location *j*, that is, $\mathcal {I}_j = \left\{ i \in \mathcal {I} \mid f\left( d_{ij}\right) > 0 \right\}$. Related is $\mathcal {I}_j^*$, which is the set of all demand assigned to the facility at location *j*. Thus, $\mathcal {I}_j^* = \{ i \in \mathcal {I}_j \mid J_i^*\ne \text {nil}\}$, for all $j \in \mathcal {J}$.

Let $\varphi _i$ denote the best coverage that demand point *i* receives in the current solution, $\varphi _i = \{c_{iJ_i^*} \mid J_i^* \ne \text {nil} \}$. In case no facility is assigned to demand *i* (i.e. ${J}_i^*$ = nil), we define $\varphi _i = 0$. Furthermore, let the variable $\varphi _j^0$ denote the total potential coverage that a facility at location *j* can add to the current solution, assuming that currently no facility is placed at candidate location *j* (i.e. $Y_j = 0$), thus $\varphi _j^0 = \sum _{i \in \mathcal {I}_j} \max \{c_{ij}-\varphi _i,0 \}$.

Using the introduced notations, we now define the Greedy algorithm for the MCLP in Algorithm 1, having as input the sets $\mathcal {I}$ and $\mathcal {J}$, the coverage matrix $\varvec{C}$ with values $c_{ij} = f(d_{ij})$, and the number of facilities to be deployed *N*.

**Algorithm 1:**
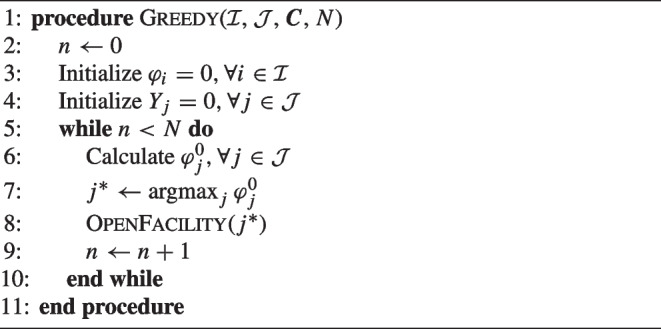
Greedy algorithm for the MCLP with partial coverage.

First, the increase in objective function $\varphi _j^0$ by opening location *j* is calculated for each candidate location, after which the location with the highest value is chosen to be opened. Then OpenFacility checks all demand that can be covered by the new facility in set $\mathcal {I}_j$ and assigns them to this new facility if their coverage is improved. Unlike the regular MCLP, using gradual coverage means that the already assigned demand may need to be reassigned to the newly added facility. Note that after opening location $j^*$, in the next loop, $\varphi _j^0$ needs to be updated accordingly only for candidate locations $j \in \bigcup _{i \in I_{j^*} } \mathcal {J}_i$.

#### Greedy randomized adaptive search procedure

To define the GRASP algorithm, let $\mathcal {S}$ denote the set of solutions found in the algorithm where initially $\mathcal {S} = \emptyset$, and in total $\theta$ solutions are constructed. Furthermore, let $s^*$ denote the best solution among all solutions and *z*(*s*) denote the objective value of a solution *s*. The generic GRASP is given in Algorithm 2.

**Algorithm 2:**
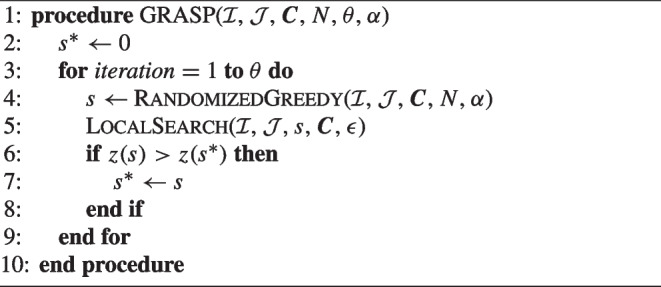
General procedure of GRASP.

During each iteration of GRASP’s construction phase RandomizedGreedy, the next element to be added is *randomly* selected from a restricted candidate list (RCL), as opposed to the deterministic selection as in Greedy. The RCL is a subset of all candidate locations where no facility has been placed yet that have a contribution to the objective value $\varphi _j^0$ above a certain threshold. It is defined as $\text {RCL} = \{j \in \mathcal {J}^c \mid \varphi _j^0 \ge \min _{k \in \mathcal {J}^c} \varphi _k^0 + \alpha (\max _{l \in \mathcal {J}^c} \varphi _l^0 - \min _{k \in \mathcal {J}^c} \varphi _k^0)\}$. The overall quality of the elements in the RCL is tuned by parameter $\alpha \in [0,1]$. Consequently, the length of the RCL may be different in each stage of adding a facility.

In the local search phase, neighborhoods are explored to find better solutions. For an incumbent solution, every possible swap of an active location $j_1 \in \{ j \in \mathcal {J}^c \mid Y_j = 1 \}$ and an inactive location $j_2 \in \{ j \in \mathcal {J}^c \mid Y_j = 0 \}$ is evaluated. Afterwards, the swap $(j_1^*, j_2^*)$ with the largest improvement in the value of the objective function is chosen and applied to the incumbent solution. This process continues until the improvement of the best swap is smaller than $\epsilon$.

## Case study

Subsection 4.1 presents our data. Subsection 4.2 describes our approach to formulating the coverage function. Finally, Subsection 4.3 discusses how we estimate the spatial probability distribution of OHCA incidences and utilize it to find and evaluate solutions.

### Data

#### Cardiac arrest and AED data

We obtained historical OHCA data from Amsterdam REsuscitation STudies (ARREST), which is an ongoing prospective registry of all OHCAs that occur in most of the municipalities of the province North Holland in the Netherlands. Data from January 1, 2006 to December 31, 2016 is included (11 years). OHCA locations are included in the form of addresses. The geocoding functionality of the geographical information system software ESRI ArcMap 10.5 was used to convert these addresses into spatial coordinates. The instances where the software indicated that the interpretation of the address was ambiguous were manually checked and corrected.

We also obtained GPS coordinates of the AED locations from the VRS HartslagNu, which are registered AEDs intended to be used by HartslagNu volunteers and can be publicly or privately owned. We included the 29 municipalities for which we have both the OHCA and the AED data. Cardiac arrests for which resuscitation was not started, or which had a non-medical cause as determined using the Utstein guidelines, were excluded. The final dataset consists of 4229 OHCAs and 1149 AEDs.

#### HartslagNu data

We received 1721 completed surveys of HartslagNu volunteers that were assigned an AED and arrived at the scene of the alert location between November 1, 2016 and December 1, 2017. From those, we found 949 unique alert locations.

**Table 1:** Euclidean distance multipliers $q_t$ fit using least squares for different modes of transportation

Mode of transportation *t*	$q_t$	Avg. Google Maps	Euclidean errors		Multiplier errors	
		distance (m)	RMSE	MAPE	RMSE	MAPE
Foot	1.383	503	228	28.2%	164	17.1%
Bicycle	1.519	555	289	33.3%	195	21.6%
Car	1.961	742	639	44.1%	503	40.0%

### Coverage decay function

The coverage decay function is a key element in the MCLP with partial coverage, modeling the decreasing effectiveness of an AED as distance to the cardiac arrest increases. We assume this function is monotonically decreasing with distance, with a cutoff at distance *r*, after which coverage remains 0. To define this function, we determine a Euclidean cutoff distance *r* (Section 4.2.2) and a shape (Section 4.2.3).

#### Distance metric

In order to calculate coverage, the distance between a cardiac arrest and candidate locations for AEDs should be calculated in an efficient manner. While many previous studies used Euclidean distance [20, 22, 24], Deakin et al. [12] showed that Euclidean distances underestimated walking distances by about 30%. Similarly, Fan et al. [50] reported that actual walking distances were nearly double the straight line distances. Furthermore, another study found walking distance multipliers between 1.4 and 1.6 for Toronto and Copenhagen, respectively [51].

In this study, we denote $q_t$ as a multiplier of the Euclidean distance for each mode of transportation to approximate the actual distances. Directly measuring distances using tools like Google Maps API is too costly for the large cardiac arrest training sets and large number of candidate locations that we use in our experiments (Section 4.3). Instead, we used Google Maps API to calculate the distances and expected travel times for 949 unique HartslagNu alert locations and their corresponding AED locations, repeated for each mode of transportation. Table 1 shows the root mean square error (RMSE) and the mean absolute percentage error (MAPE) for both Euclidean distances (i.e. $q_t=1$) and the adjusted distances using the multipliers $q_t$. Car has the highest multiplier, the highest MAPE, and also the lowest impact of using a multiplier. These findings align with those reported in the literature [12, 51].

#### Cutoff point

From 1721 completed HartSlagNu surveys, we found that 22% of the volunteers traveled on foot, 33% by bicycle, and 45% by car. We determined a cutoff distance $r_t$ for each mode of transportation using the following approach:

1. Estimate average travelling speed $s_t$ for each mode of transportation.
2. Define the time interval in which coverage would decrease from 1 to 0.
3. Convert this time interval to a cutoff distance $r_t$, using $s_t$ and $q_t$.

Travel speeds for bicycling and driving were estimated using Google Maps, as outlined in Section 4.2.1. For walking, we assumed a brisk walking pace of 8 km/h, consistent with findings from Jonsson et al.[52] in Sweden and previously used by Chan et al. [20].

**Fig. 1:**
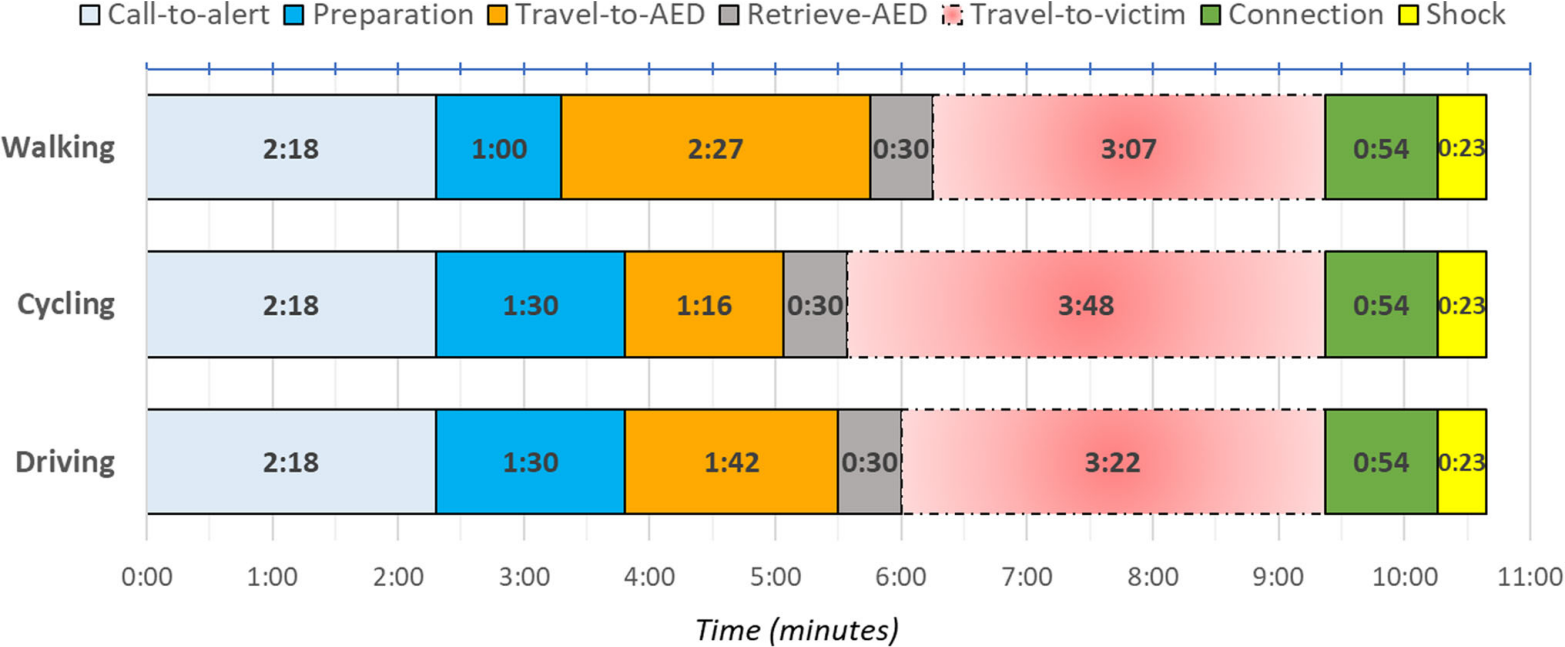
Timeline (minutes) of an average volunteer, depending on the mode of transportation. The travel-to-victim time depends on AED locations, and is here chosen as the maximum value that this activity could take in reference to median EMS shock time of 10:39 minutes

To define a time interval, we developed a timeline detailing the activities of a dispatched volunteer responding to a HartslagNu alert. The aim was to determine the average earliest arrival time at the scene. We estimated the average duration of each activity based on our data or relevant literature, adjusting for the mode of transportation, which resulted in three distinct timelines.

- Call-to-alert: Time between calling the emergency number and the activation of HartslagNu, based on historic data. (2:18 minutes)
- Preparation: Time for volunteers to process the alert and prepare. An additional 30 seconds is added for those using bicycles or cars (1:00 or 1:30 minutes)
- Travel-to-AED: Time to travel from the volunteer’s location to the assigned AED. Estimated by finding the average minimum Euclidean distance between the volunteer and the AED per alert, which was 236m, and adjusted for travel speed ${s_t}$ and distance multiplier $q_t$. ($\frac{q_t236}{s_t}$: 2:25 minutes for walking, 1:16 minutes for cycling, and 1:42 minutes for driving)
- Retrieve-AED: Time estimated for retrieving the AED, considering some are placed in secured cabinets. (0:30 minutes)
- Travel-to-victim: Time to travel from the location of the AED to the victim, which will vary depending on AED placement and would therefore be a result of the model.
- Connection: Time to connect an AED to the victim. Gundry et al. [53] showed that on average professionals connected an AED and defibrillated the patient in 67 seconds. We subtracted 23 seconds to remove the shock part and added 10 seconds for preparation at the scene based on expert opinions. (0:54 minutes)
- Shock: Time to deliver the first shock after the AED is connected, obtained from the ARREST data set. (0:23 minutes)

Figure 1 displays the timeline and indicates that the fastest possible time-to-shock would be 6:51 minutes, by assuming no travel-to-victim time and transportation by bicycle. In general, an AED retrieved by a volunteer only provides value if it is connected before EMS arrives. Therefore, to determine a time interval in which the volunteer could add value, we refer to the median EMS shock time of 10:39 minutes [16] as the time at which coverage would be 0. Table 2 shows the travel speeds, the time intervals, and the corresponding Euclidean cutoff distances for each mode of transportation, rounded up to the nearest 10m.

**Table 2:** Parameters used to determine the cutoff points $r_t$ for the coverage functions

Mode of travel *t*	$s_t$	Time interval	$r_t$
Foot	8.0 km/h	3:07 minutes	310 meters
Bicycle	16.9 km/h	3:48 minutes	710 meters
Car	16.4 km/h	3:22 minutes	470 meters

#### Shape

We used a linear coverage decay function for each of the modes of transportation. Our timeline indicates that the earliest time-to-shock would be 6:51 (*t* = bicycle, with zero travel-to-victim time). Although our timeline was based on averages, in practice, only a small minority of cardiac arrests receive a shock within 6 minutes, even after introduction of a volunteer responder system ([15, 54]). The survival curve of Waalewijn et al. [55] shows that the slight curve between 6:51 and 10:39 minutes (median EMS shock time) can be well approximated by a linear function. Previous research by Chan et al. [20] used exponential coverage decay, mimicking the exponential decrease in survival as a function of time. Their assumptions are based on the whole survival curve, which starts at 0 minutes and may go well beyond 15 minutes. Instead, we focus on the time segment in which volunteer defibrillation realistically could occur, given the inherent delays in volunteer response and EMS arrival.

**Fig. 2:**
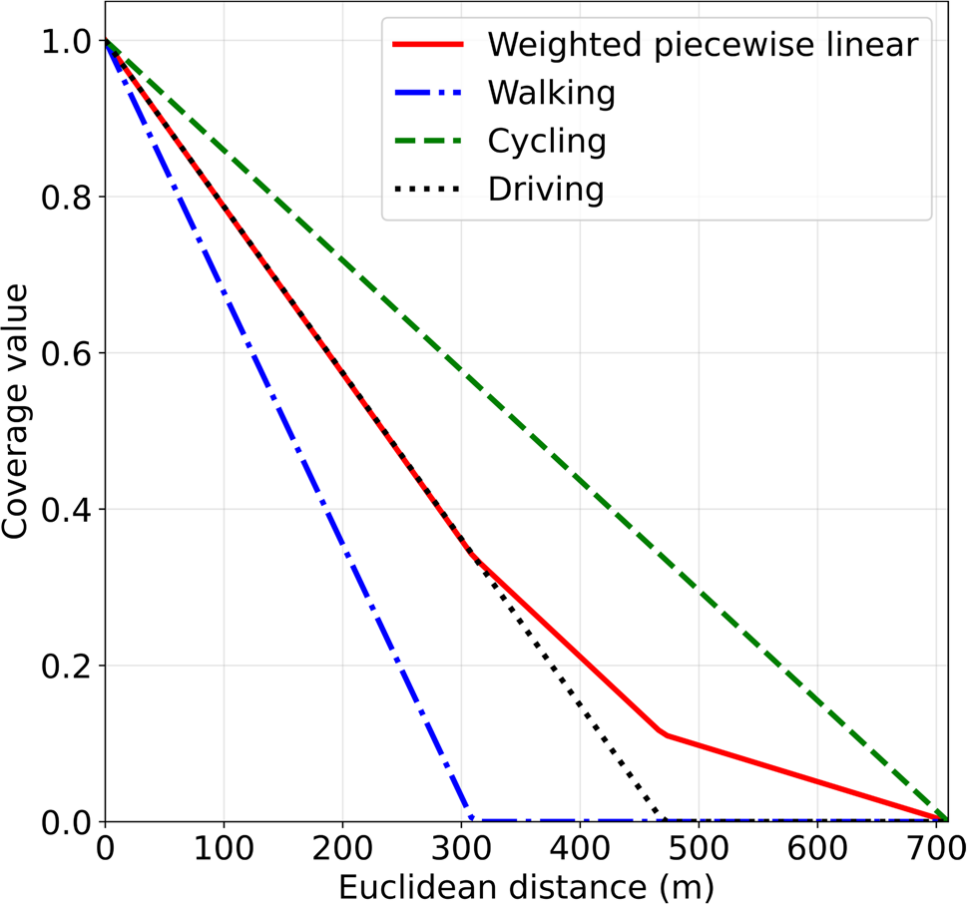
The piecewise linear coverage function (solid line) obtained by taking a weighted combination of the coverage functions for each mode of transportation

**Fig. 3:**
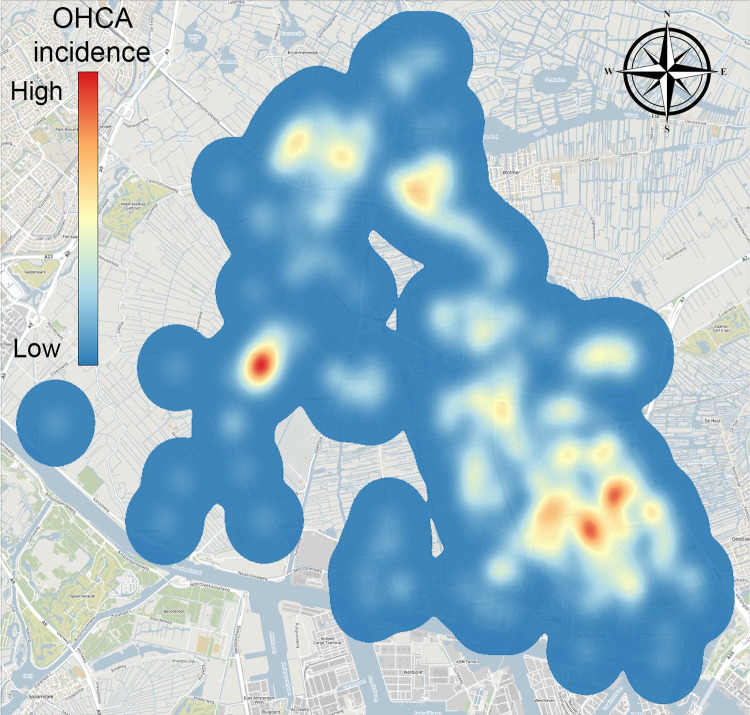
KDE of the municipality of Zaanstad (Background: ©OpenStreetMap contributors, CC BY-SA)

Using the cutoff points $r_t$ determined in Table 2, Fig. 2 shows the coverage function for each mode of transportation and the resulting weighted coverage function $f(d) = \sum _{t \in T} w_t\max \{1 - \frac{d}{r_t}, 0\}$. This function is piecewise linear with breakpoints at the cutoff distances $r_t$.

### Generating cardiac arrest locations

We transformed historic cardiac arrest locations into a spatial distribution of cardiac arrest risk by applying bivariate Kernel Density Estimation (KDE) with Gaussian kernels. The nonparametric method of Botev et al. [56] was used to determine an appropriate bandwidth. The application of KDE in AED optimization is validated in the Appendix A. Figure 3 shows the KDE of the municipality of Zaanstad.

After obtaining this spatial distribution of cardiac arrest risk, we can sample new locations accordingly. To properly assess the performance of our solutions, we sample a large training set of cardiac arrest $\mathcal {I}^{train}$ and a larger evaluation set of cardiac arrest $\mathcal {I}_k^{eval}$. The training set will be input to the methods, while afterwards the evaluation set is used to assess ‘out-of-sample’ performance of the chosen locations to deploy AEDs.

Most studies focusing on modeling spatial risk of out-of-hospital cardiac arrest (OHCA) employed models that consolidated data into spatial cells [42, 57–64]. Spatial analysis techniques, such as Getis-Ord Gi* statistic, were employed to identify high-risk census tracts [57–59]. Another strategy involved the utilization of a Bayesian model incorporating parameters for spatial (and temporal) heterogeneity, space-time interactions, and demographic covariates [42, 60–62]. However, usage of discrete models, by assuming uniform incidence across spatial cells, may lead to abrupt transitions in incidence rates, both within and around the borders of these cells. It is evident that the definition of spatial cells significantly influences the analyses and subsequent results.

KDE offers a notable advantage in that it provides a continuous estimate without necessitating the delineation of the study region into predefined spatial cells. Consequently, the outcomes are not influenced by the boundaries of these spatial cells or administrative areas. KDE applies a continuous density function at each observed data point with a specified bandwidth, which is proportional to the standard deviation of the density function, resulting in an aggregated density function [65]. KDE has been used to estimate the spatial distribution of cardiac arrest risk before [20, 50, 66–68].

## Experimental design & results

In this section, we show the results from applying our models across 29 municipalities. First, Section 5.1 explains the values chosen for the GRASP parameter $\alpha$ and the determination of the evaluation set size. Section 5.2 examines how the size of the problem instance impacts the results, and compares the performance of BIP, GRASP, and the Greedy algorithm on a large problem instance. Section 5.3 shows the performance of GRASP on the relocation problem and Section 5.4 shows the effect of deploying additional AEDs in addition to the existing AED locations. Lastly, Section 5.5 explores variations in the assumed shape of the coverage decay function by comparing different coverage functions.

First we define how performance of AED locations is measured on the evaluation set of cardiac arrests. Recall that $Y_j$ is 1 if location $j\in \mathcal {J}$ is opened and 0 otherwise. Let $Y^*_j$ indicate if location $j \in \mathcal {J}$ is opened in the solution obtained using one of the solution methods, then $J^* = \{ j \in J \vert Y^*_j = 1 \}$ is the set of locations with an AED. Note that since $J = J^e \cup J^c$, $J^*$ includes existing AEDs if $J^e \ne \emptyset$. Then we define the performance measure

3 $$\begin{aligned} z_m = \frac{100\%}{|I_k^{eval} |}\sum _{k \in \{1,\dots ,K\}} \sum _{i \in I_k^{eval}} \max _{j \in J^*} \sum _{t \in T}w_tf^t(d_{ij}) \end{aligned}$$

to be the average coverage across all OHCAs in the evaluation set $I_k^{eval}$, for a municipality *m*. We multiply the coverage values by 100% to make results easier to read. The average performance across all municipalities in the data is calculated by weighting the results of each municipality by its number of historic cardiac arrests $\mu _m$

4 $$\begin{aligned} z = \frac{100\%}{\sum _{m \in M}\mu _m}\sum _{m \in M}{\mu _m z_m} \end{aligned}$$

The heuristics are implemented in Python 3.9.7 and compiled by Numba 0.56.0 (a high performance compiler). The BIP problem is solved with Gurobi version 9.5.0. The experiments are executed on a Windows laptop with a 1.90 GHz i7-8665U quad-core processor and 16 GB of RAM. Random number seeds are fixed for the sake of reproducibility.

### Parameters

#### GRASP parameters

GRASP requires specifying values for the parameter $\alpha$ for the restricted candidate list in the construction phase and the stopping criterion $\epsilon$ for the local search procedure. We take a sufficiently small $\epsilon = 5e^{-6}$.

For $\alpha$, a possible strategy is to use multiple values to find a greater diversity of solutions and to rely less on parameter tuning [44]. We aim to use a general scheme that allows for exploration of the solution space for all our different municipalities and problem sizes. Therefore, we initialize $\alpha = 0.95$ and decrease $\alpha$ by 0.01 at the end of each iteration (until $\alpha =0$), to gradually open up the solution space.

#### Evaluation set

After finding a solution using the training data, we assess its performance on an evaluation set of cardiac arrests, representing unseen data. The size of the evaluation set is expressed in years of expected number of cardiac arrests in that municipality, i.e. $\left\lceil {\frac{\mu _m}{11}}\right\rceil$ cardiac arrests per year. By increasing the size of the evaluation set, we aim to better approximate the KDE.

**Fig. 4:**
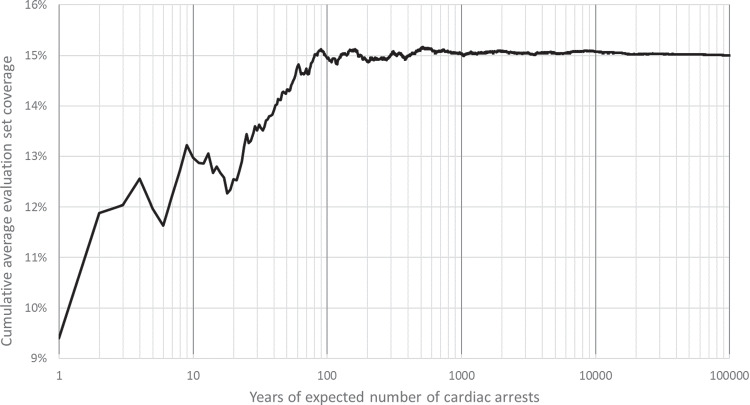
Cumulative average coverage for the size of the evaluation set (expressed in years of expected number of cardiac arrests), calculated based on existing AEDs in the municipality of Zaanstad

Figure 4 shows that as the size of the evaluation set increases, the cumulative average coverage stabilizes. Coverage calculations were performed for existing AED locations in the municipality of Zaanstad. Given that sampling new locations and assessing coverage is relatively quick, we conservatively set the size of the evaluation set to represent 50000 years of cardiac arrests for all municipalities.

### Problem size

A granular set of candidate locations allows for finding better performing solutions. We determined that a 100m distance between neighboring candidate locations is suitable for practical applications. A smaller distance might be overly precise, given the improbability that an AED can be deployed exactly at the specified location. We also pre-eliminate candidate locations that offer no coverage to any historical OHCA incidents.

Since the KDE is approximated by the simulated training set of locations, it is important to maximize the number of demand points while keeping the problem tractable. Insufficient sampling can lead to an inaccurate representation of the spatial distribution, potentially degrading performance on the evaluation set.

Table 3 presents the characteristics of the 29 municipalities. The OHCA-to-AED ratio ranges between 1.2 and 50 (IQR: 2.2-6.6), reflecting a diverse set of baseline scenarios. The number of candidate locations also vary widely, influenced by the municipality’s size and the spatial distribution of OHCA.

**Table 3:** Description of the municipalities

Municipality	Existing AEDs	Number of OHCAs $\mu _m$	OHCA-to-AED ratio	Candidate locations
Aalsmeer	3	99	33.0	5600
Alkmaar	118	392	3.3	14452
Beemster	28	34	1.2	7793
Bergen	42	171	4.1	10010
Castricum	49	150	3.1	6328
Den Helder	50	253	5.1	6351
Diemen	2	100	50.0	2183
Drechterland	26	55	2.1	8047
Edam-Volendam	84	146	1.7	7819
Enkhuizen	24	76	3.2	2381
Heerhugowaard	39	181	4.6	5525
Heiloo	16	97	6.1	4103
Hollands Kroon	80	199	2.5	30387
Hoorn	84	222	2.6	4595
Koggenland	56	77	1.4	9680
Landsmeer	5	32	6.4	3293
Langedijk	23	65	2.8	5915
Medemblik	80	167	2.1	16188
Oostzaan	1	40	40.0	2195
Opmeer	26	49	1.9	6582
Ouder-Amstel	2	62	31.0	5211
Purmerend	47	312	6.6	4237
Schagen	91	209	2.3	21602
Stede Broec	16	62	3.9	2276
Texel	65	108	1.7	16603
Uithoorn	7	105	15.0	3601
Waterland	35	76	2.2	8571
Wormerland	6	74	12.3	5749
Zaanstad	44	616	14.0	11989

Candidate locations are based on a 100m uniform grid. Number of OHCAs are from 2006 to 2016

**Table 4:** Comparison of the performance of BIP, GRASP, and Greedy in problem instances with 30387 candidate locations and varying number of demand points (Hollands Kroon)

Method	Demand points	Training set	Evaluation set	CPU time (hr)	Optimality gap
BIP	5000	47.34%	44.77%	0.51	<0.01%
	10000	46.86%	45.07%	2.93	<0.01%
	20000	46.30%	45.28%	28.07	<0.01%
	30000	46.05%	45.34%	41.81	0.21%
	40000	Memory error			
	50000	Memory error			
GRASP	5000	47.27%	44.73%	1.96	
	10000	46.77%	45.05%	2.00	
	20000	46.18%	45.30%	2.00	
	30000	45.96%	45.31%	1.90	
	40000	46.04%	45.39%	1.88	
	50000	45.78%	45.41%	1.82	
Greedy	5000	46.19%	43.93%	6.42e-4	
	10000	46.08%	44.47%	2.21e-4	
	20000	45.34%	44.68%	4.99e-4	
	30000	45.29%	44.74%	7.55e-4	
	40000	45.31%	44.77%	9.90e-4	
	50000	45.07%	44.79%	1.25e-3

**Table 5:** Current and relocation performance for each municipality on the evaluation set

Municipality	Current	Relocation	Relative	CPU time	Coverage per year percentile				
	performance	performance	improvement	(hr)	10th	25th	50th	75th	90th
Aalsmeer	6.27%	13.49%	115.0%	2.04	3.98%	7.85%	12.71%	18.18%	23.69%
Alkmaar	51.17%	60.80%	18.8%	1.36	56.20%	58.45%	60.90%	63.26%	65.30%
Beemster	34.63%	49.35%	42.5%	1.96	28.27%	39.47%	50.11%	60.28%	69.57%
Bergen	41.14%	49.31%	19.9%	2.06	40.91%	45.00%	49.39%	53.75%	57.60%
Castricum	49.21%	59.36%	20.6%	1.94	51.91%	55.66%	59.57%	63.34%	66.66%
Den Helder	43.54%	55.05%	26.5%	2.08	48.83%	51.89%	55.15%	58.24%	60.99%
Diemen	16.08%	23.18%	44.1%	2.04	12.04%	16.94%	22.68%	28.86%	34.74%
Drechterland	38.92%	54.01%	38.8%	1.99	39.32%	46.87%	54.47%	61.84%	68.51%
Edam-Volendam	59.63%	70.33%	17.9%	2.10	64.10%	67.34%	70.66%	73.73%	76.34%
Enkhuizen	51.71%	60.96%	17.9%	1.95	49.77%	55.72%	61.49%	66.77%	71.42%
Heerhugowaard	38.15%	51.42%	34.8%	1.75	43.88%	47.51%	51.50%	55.38%	58.77%
Heiloo	35.99%	43.98%	22.2%	1.94	31.72%	37.82%	44.12%	50.29%	56.07%
Hollands Kroon	32.65%	45.41%	39.1%	1.81	36.75%	40.92%	45.46%	49.96%	53.96%
Hoorn	53.69%	65.00%	21.1%	2.14	60.05%	62.49%	65.13%	67.60%	69.78%
Koggenland	40.88%	63.00%	54.1%	2.00	52.47%	57.82%	63.46%	68.72%	73.20%
Landsmeer	22.41%	31.32%	39.8%	2.03	10.06%	20.28%	30.74%	41.70%	52.19%
Langedijk	38.13%	48.01%	25.9%	2.01	33.21%	40.63%	48.36%	55.77%	62.81%
Medemblik	40.05%	53.75%	34.2%	2.03	45.47%	49.61%	53.95%	58.18%	62.01%
Oostzaan	16.57%	16.76%	1.2%	2.04	2.06%	6.47%	14.67%	24.25%	33.90%
Opmeer	42.13%	51.38%	22.0%	2.03	34.10%	43.32%	51.98%	60.28%	68.45%
Ouder-Amstel	8.02%	15.93%	98.6%	2.03	3.08%	8.05%	14.62%	22.13%	29.74%
Purmerend	47.31%	55.97%	18.3%	2.05	50.92%	53.36%	55.99%	58.63%	60.96%
Schagen	38.27%	47.57%	24.3%	2.01	39.08%	43.21%	47.69%	52.09%	55.98%
Stede Broec	36.77%	49.48%	34.6%	1.99	35.90%	42.82%	49.74%	56.42%	62.66%
Texel	38.39%	50.99%	32.8%	2.06	39.96%	45.43%	51.17%	56.80%	61.92%
Uithoorn	15.47%	36.60%	136.6%	2.04	25.02%	30.52%	36.59%	42.62%	48.31%
Waterland	38.61%	54.83%	42.0%	1.91	42.77%	48.96%	55.13%	61.10%	66.73%
Wormerland	14.74%	32.11%	117.8%	2.04	16.87%	24.13%	31.83%	39.92%	47.62%
Zaanstad	15.02%	41.38%	175.5%	1.80	36.95%	39.06%	41.40%	43.70%	45.78%

Results are obtained using GRASP with 50000 demand points and 100m candidate location grid, with a wall time limit of 2 hours

#### Heuristics compared to exact method

To compare the heuristics with the BIP, we first solve the relocation problem for the municipality with the largest number of candidate locations, which according to Table 3 is Hollands Kroon. In addition, our aim was to determine at what point the memory requirements for solving the BIP problem exceed the available computer memory limits. We apply the heuristics and solve the BIP problem for identical problem instances. Only the best solution GRASP found for the training set is evaluated on the evaluation set.

For each problem instance, a time limit of 24 hours (wall-clock time) was imposed for solving the BIP problem. GRASP was allotted 2 hours for smaller instances (5000, 10000, 20000) and 6 hours for larger ones (30000, 40000, 50000). Gurobi was able to solve the BIP problem for an instance of 30000 demand points before running into memory errors, but with a 0.21% optimality gap (Table 4). Even with more computer memory, it is reasonable to assume that computation time would explode for even larger problem instances, as CPU time was nearly 42 hours for 30000 demand points.

We observe that the performance of the solutions found using GRASP is very similar to those found using the BIP, on both the training and evaluation set (Table 4). GRASP’s optimality gaps ($\frac{\text {obj}_{\text {BIP}}-\text {obj}_{\text {GRASP}}}{\text {obj}_{\text {BIP}}}\%$) on the training sets were 0.16%, 0.19%, 0.26%, and 0.18% for 5000, 10000, 20000, and 30000 demand points, respectively. For the largest problem size Gurobi was able to solve the BIP problem, i.e. 30000 demand points, GRASP found a solution with evaluation performance of 45.31% compared to 45.34% obtained from the BIP problem.

The results illustrate that increasing the number of demand points is important. The difference between evaluation set coverage of 10000 points (45.07%, BIP) and 50000 points (45.41%, GRASP) is substantial (Table 4). We stopped increasing demand points after 50000 because improvements in the evaluation performance became sufficiently small and the iterations of GRASP become slower. Also, we already surpassed the largest problem instance for which Gurobi was able to solve the BIP problem. Note that average training set coverage tends to decrease as the training set size increases, because there are more demand points to consider in optimizing the locations. As the number of demand points increases, the gap between training and evaluation performance decreases.

While the BIP and GRASP led to substantially better solutions than Greedy, Greedy’s performance was remarkable for a simple and very fast heuristic. Even the largest problem instance with 50000 demand points took less than 5 seconds of CPU time. In addition, Greedy’s performance gives perspective to the improvement GRASP makes.

In Sections 5.3 and 5.4 we analyze the relocation and addition of AEDs for the 29 municipalities with solutions found using GRASP. We use GRASP because the results in Table 4 show that GRASP can consistently find solutions that are very close to the BIP problems’ solutions, in much less time. In addition we were able to solve a larger problem instance, increasing the potential of solutions, thus from now on we use 50000 demand points for each municipality.

### Relocating existing AEDs

In this section we analyze the relocation problem using GRASP (Table 5). For each municipality, we generated a problem instance with 50000 cardiac arrests and 100m between neighboring candidate locations. GRASP was run for 2 hours. Current performance, relocation performance, and coverage per year in Table 5 are all measured on the evaluation set. Coverage per year is calculated by sampling 50000 new sets of cardiac arrests with their size sampled from the empirical distribution of the number of cardiac arrests in a year.

From Table 5 we observe that the performance of the existing AED locations vary greatly across municipalities. Current coverage ranges from as low as 6.27% (Aalsmeer, 3 AEDs) to as high as 59.63% (Edam-Volendam, 84 AEDs), largely attributable to the number of AEDs. Interestingly, municipalities with comparable numbers of AEDs *and* OHCAs can exhibit significant differences in performance. For instance, Edam-Volendam and Medemblik have coverage of 59.63% and 40.05%, respectively, despite similar OHCA-to-AED ratios of 1.7 and 2.1.

**Fig. 5:**
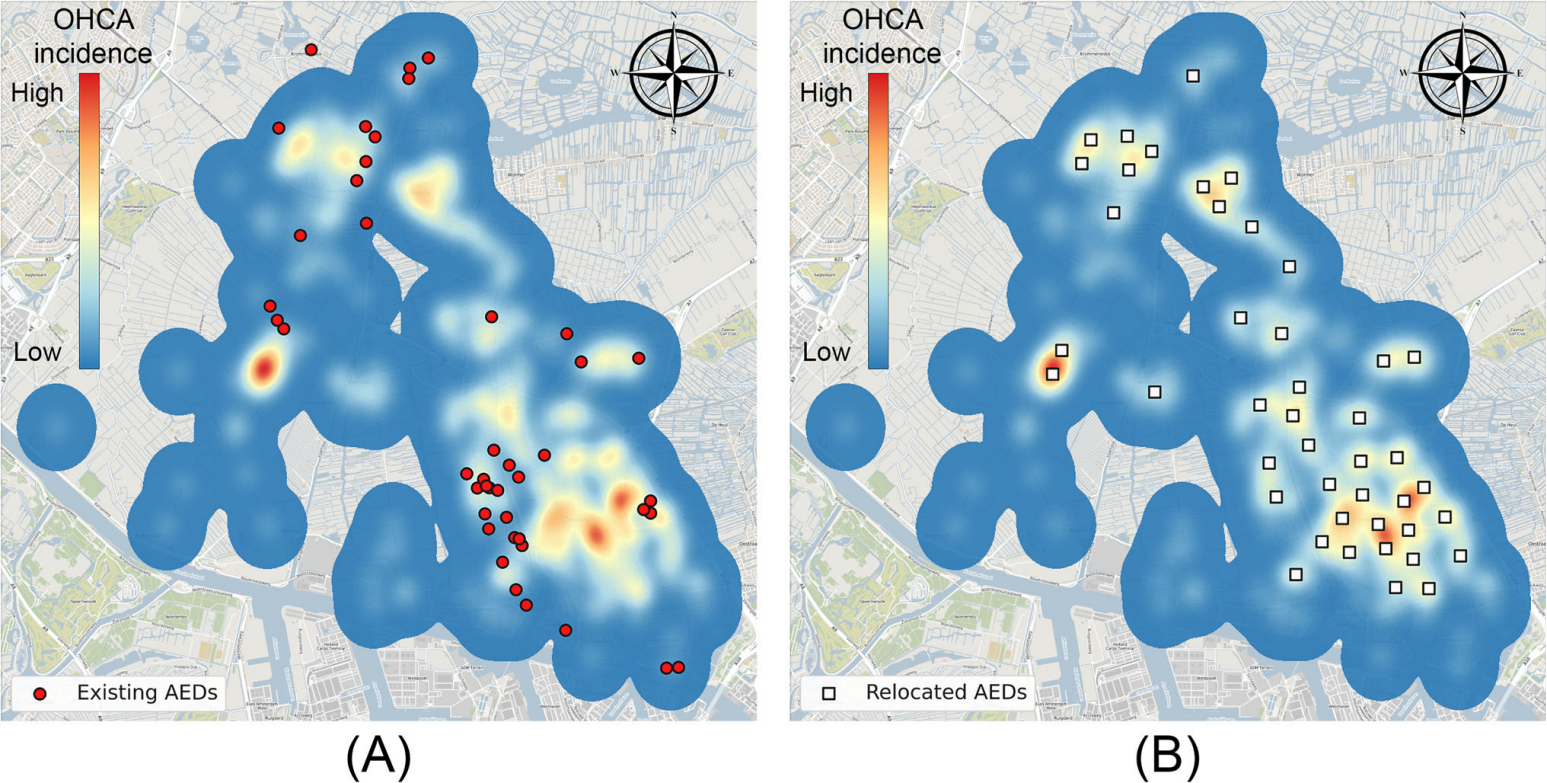
Existing AEDs **(A)** and relocated AEDs using GRASP **(B)** for the municipality of Zaanstad (Background: ©OpenStreetMap contributors, CC BY-SA)

When relocating all the AEDs within a municipality, the weighted average performance across municipalities increased from 36.14% to 49.53%. The extent of improvement varied depending on the quality of the existing locations, the number of AEDs, and the spatial distribution of cardiac arrests. It is important to contextualize these gains, particularly in areas where baseline performance was already high, making significant improvements more challenging.

**Fig. 6:**
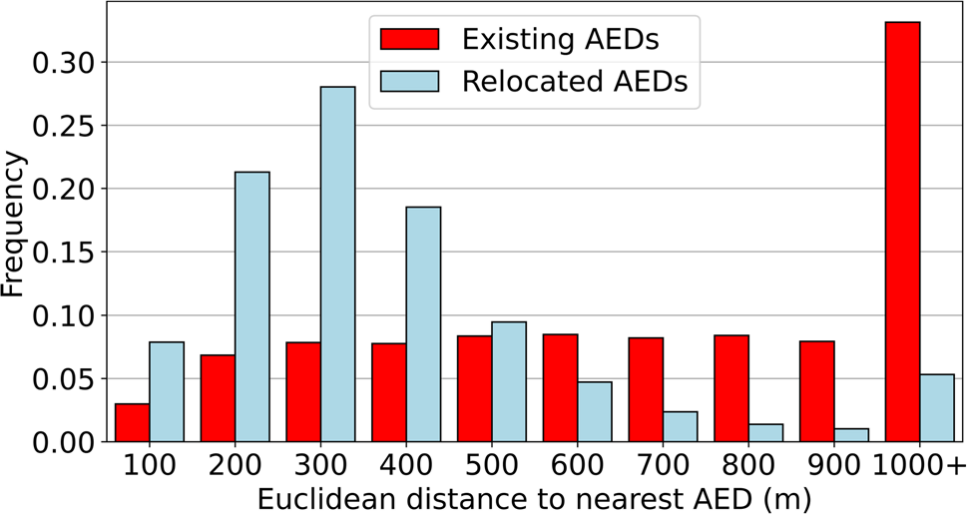
Distance distribution to nearest AED in Zaanstad for current AED locations (*left bars*) and for relocated AEDs (*right bars*), calculated for OHCAs in the evaluation set

The greatest relative improvements were observed in Zaanstad (175.5%, 44 AEDs), Uithoorn (136.6%, 7 AEDs), and Wormerland (117.8%, 6 AEDs). In Zaanstad, the existing AED placements did not align well with OHCA occurrences (Fig. 5). Relocated AEDs provided better coverage, especially in hotspots, and were more evenly distributed across the municipality (Fig. 5). Additionally, the relocation dramatically improved the proximity of AEDs to OHCAs. Initially, many OHCAs were over 1 km away from an AED, but after relocation, over half were within 400m (Fig. 6).

Oostzaan saw the least improvement, primarily because it only has one AED. Purmerend achieved a relative improvement of 18.3%, with 47 AEDs, suggesting that its existing AED locations are already quite effective compared to those in other municipalities.

Additionally, the variation in coverage per year is considerable, as indicated by the corresponding percentiles. While deploying more AEDs reduces this variability, the uncertainty associated with the unpredictable locations of future OHCAs remains a significant challenge.

**Table 6:** Performance of adding AEDs in addition to existing AEDs

	Number of additional AEDs				
Municipality	0	5	10	20	40
Aalsmeer	6.27%	23.43%	32.60%	43.69%	55.16%
Alkmaar	51.17%	53.62%	55.21%	57.74%	61.46%
Beemster	34.63%	41.72%	46.96%	54.03%	60.72%
Bergen	41.14%	46.21%	49.03%	53.44%	59.06%
Castricum	49.21%	53.98%	57.10%	61.41%	66.77%
Den Helder	43.54%	48.28%	51.21%	55.68%	61.63%
Diemen	16.08%	43.81%	53.55%	63.61%	72.54%
Drechterland	38.92%	46.23%	51.38%	59.85%	67.09%
Edam-Volendam	59.63%	62.96%	64.72%	67.72%	71.70%
Enkhuizen	51.71%	59.27%	62.96%	68.11%	73.65%
Heerhugowaard	38.15%	45.20%	49.09%	54.42%	61.11%
Heiloo	35.99%	45.22%	50.68%	57.28%	64.49%
Hollands Kroon	32.65%	36.26%	38.43%	41.82%	46.49%
Hoorn	53.69%	57.25%	59.55%	62.97%	67.26%
Koggenland	40.88%	46.41%	50.30%	56.27%	64.09%
Landsmeer	22.41%	38.81%	47.37%	57.02%	66.54%
Langedijk	38.13%	46.17%	50.29%	55.68%	63.05%
Medemblik	40.05%	44.45%	47.09%	50.62%	55.59%
Oostzaan	16.57%	46.07%	57.27%	67.21%	75.44%
Opmeer	42.13%	47.42%	51.60%	57.93%	64.49%
Ouder-Amstel	8.02%	30.90%	40.88%	51.67%	62.31%
Purmerend	47.31%	52.11%	55.15%	59.16%	64.76%
Schagen	38.27%	40.79%	42.53%	45.30%	49.52%
Stede Broec	36.77%	49.05%	54.86%	61.70%	69.14%
Texel	38.39%	41.80%	44.25%	48.01%	54.08%
Uithoorn	15.47%	36.75%	48.27%	57.77%	67.70%
Waterland	38.61%	46.39%	51.35%	57.18%	63.91%
Wormerland	14.74%	38.04%	48.02%	59.09%	69.62%
Zaanstad	15.02%	24.09%	30.06%	37.55%	47.18%
Overall	36.14%	43.69%	47.92%	53.31%	59.87%

Results were obtained using GRASP with 50000 demand points and 100m candidate location grid

### Deploying additional AEDs

Relocating all existing AEDs provides an indication of the effectiveness of current locations and establishes what improvements are possible with existing resources. However, since many AEDs are privately owned but made publicly available, actual relocation is often not feasible. Consequently, we explored the impact of deploying additional AEDs while keeping the existing AEDs where they are. We ran GRASP for half an hour to place 5 or 10 additional AEDs, one hour for 20 AEDs, and two hours for 40 AEDs.

**Fig. 7:**
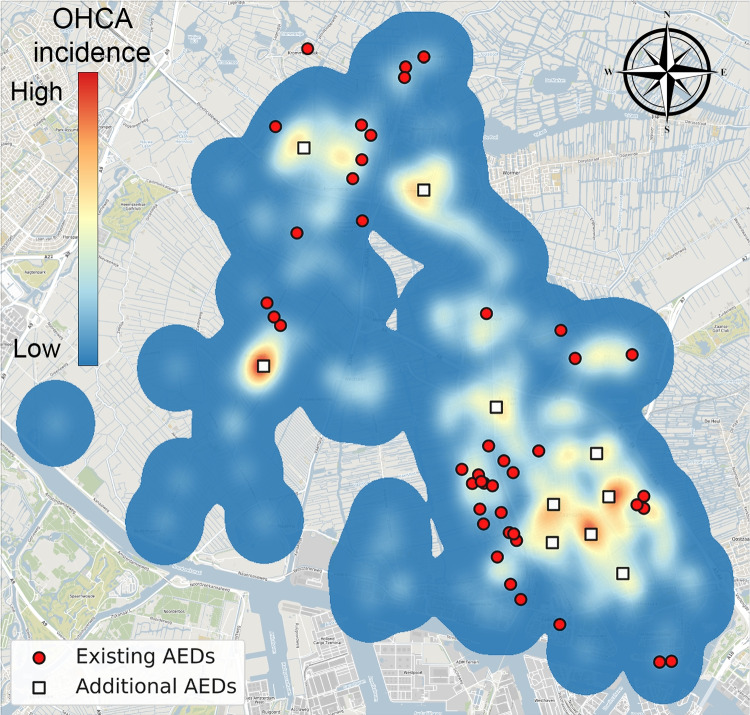
Locations of existing AEDs and proposed locations of 10 additional AEDs (GRASP) visualized on top of the KDE of the municipality of Zaanstad (Background: ©OpenStreetMap contributors, CC BY-SA)

Strategic placement of just 5 or 10 additional AEDs can significantly improve baseline coverage in most municipalities (see Table 6). For instance, Fig. 7 illustrates how placing 10 additional AEDs in Zaanstad, in addition to the 44 currently placed, effectively doubles the coverage. In municipalities like Alkmaar or Edam-Volendam, where coverage is already high, the relative improvement from additional AEDs is smaller. Notably, while both Alkmaar and Enkhuizen started with similar coverage levels, adding 40 AEDs increased their coverage to 61.46% and 73.65%, respectively.

**Fig. 8:**
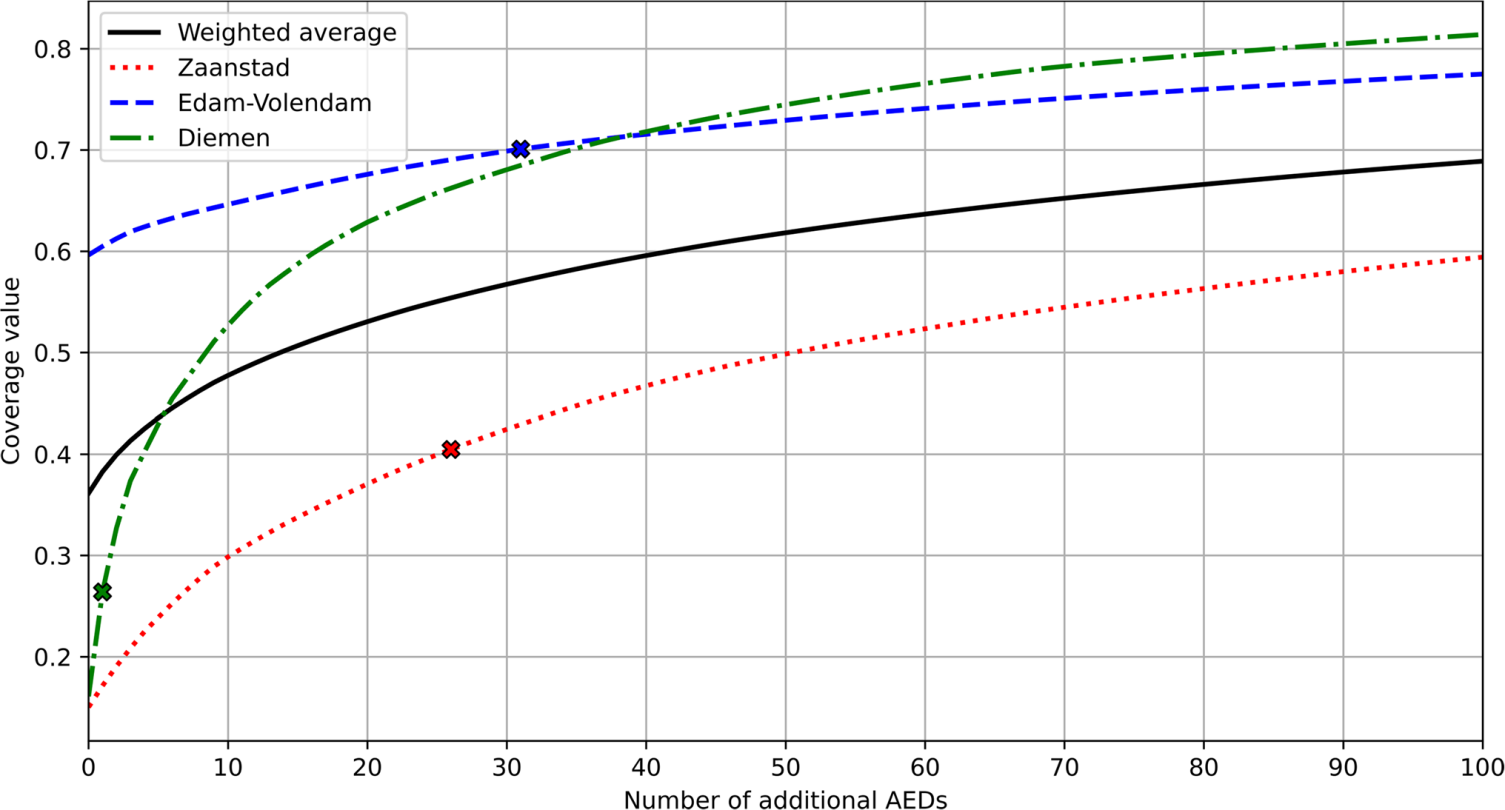
The benefits of deploying additional AEDs in various municipalities. The solid line indicates the weighted average across all 29 municipalities. The crosses indicate how many additional AEDs are necessary to match performance of relocating all existing ones

Figure 8 shows the marginal benefit curves for adding AEDs in various municipalities, as determined by GRASP. These curves illustrate that even when starting from a similar level of performance, the impact of deploying the same number of additional AEDs can vary significantly between municipalities. The crosses on the curves represent the performance achieved by relocating existing AEDs, indicating the number of additional AEDs required to match this relocation performance. Overall, approximately 428 additional AEDs (representing a 37% increase) would be necessary to achieve equivalent performance across all municipalities through additions alone.

### Sensitivity analysis of coverage decay function

**Fig. 9:**
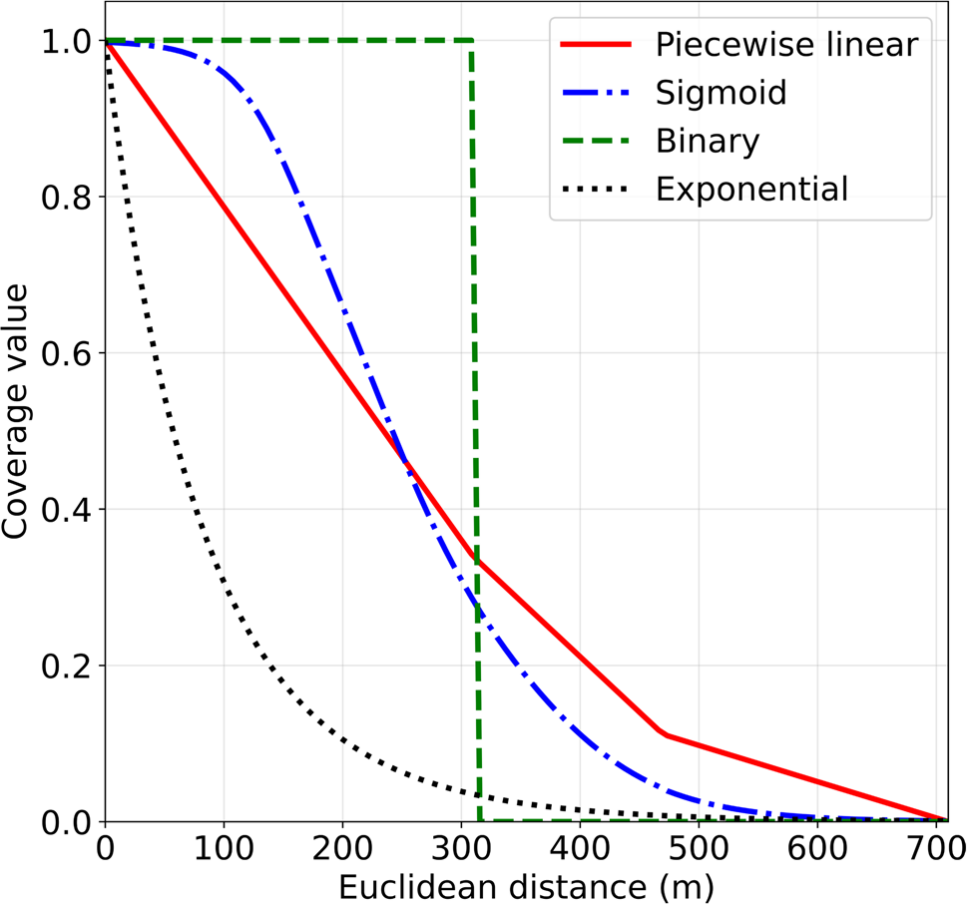
Coverage functions used for sensitivity analysis. Piecewise linear: $f_{\text {pl}}(d) = \sum _{t \in T} w_t\max \{1 - \frac{d}{r_t}, 0\}$. Sigmoid: $f_{\text {sigmoid}}(d) = \sum _{t \in T}\frac{w_t}{1+\exp (12r_t-6)}$. Binary: 1 between 0m and 310m, 0 otherwise. Exponential: $f_{\text {exponential}}(d) = \sum _{t \in T}w_t \mathrm e^{-\beta _t d}$ with coefficients $\beta _{\text {walking}}=0.02$, $\beta _{\text {cycling}}=0.0085$, and $\beta _{\text {driving}}=0.0012$

We assessed the impact of different coverage function shapes, including a binary, a weighted exponential, and a weighted sigmoid function, alongside the piecewise linear function detailed in Section 4 (see Fig. 9). For the binary function, we used a cutoff distance of 310m (from Table 2), because previous studies often only considered pedestrians for binary coverage. We defined the exponential coverage function for each mode of transportation to be $\mathrm e^{-\beta _t d}$, with *d* being the distance in meters. The coefficients $\beta _{\text {walking}}=0.02$, $\beta _{\text {cycling}}=0.0085$, and $\beta _{\text {driving}}=0.0012$ are chosen so that the coverage is close to 0 at the cutoff distance $r_t$. The weighted exponential coverage function is then $f_{\text {exponential}}(d) = \sum _{t \in T}w_t \mathrm e^{-\beta _t d}$. The sigmoid function for each mode of transportation *t* is defined as $\frac{1}{1+\exp (12r_t-6)}$ and the weighted sigmoid coverage function $f_{\text {sigmoid}}(d) = \sum _{t \in T}\frac{w_t}{1+\exp (12r_t-6)}$.

**Table 7:** Performance of AED locations obtained by an assumed coverage function, measured by the true coverage function

Coverage function		True			
		Piecewise linear	Sigmoid	Binary	Exponential
Assumed	Piecewise linear	41.34% (100.00%)	43.03% (99.23%)	59.56% (98.42%)	9.90% (95.70%)
	Sigmoid	41.11% (99.46%)	43.36% (100.00%)	59.68% (98.62%)	10.14% (98.05%)
	Binary	40.59% (98.20%)	42.53% (98.09%)	60.52% (100.00%)	9.70% (93.76%)
	Exponential	38.99% (94.32%)	41.80% (96.39%)	55.16% (91.15%)	10.35% (100.00%)

The percentages in parentheses indicate the objective value of the assumed coverage function relative to the objective value when the assumed and true coverage functions are the same

For each coverage function, GRASP was run for 2 hours to obtain relocated AED locations in Zaanstad. Afterwards, the performance of these AED locations was evaluated on the evaluation set. Table 7 presents the results when each coverage function is assumed to be correct, versus the actual performance when a different function represents reality. Values along the diagonal represent scenarios where the assumed coverage function matches the true underlying shape, serving as reference points for comparison.

We based our assumption of linear coverage functions on the segment of the OHCA survival curve where volunteer defibrillation is most likely. If any of the four coverage functions could be true however, the sigmoid shape emerges as the most robust choice, performing well across all other coverage scenarios, though its advantage over the piecewise linear function is minimal. The exponential function, characterized by a rapid decay in coverage values, yields significantly lower average coverage in comparison.

## Discussion

We modeled an AED location problem with volunteer responders utilizing different modes of transportation as an MCLP with multiple decaying coverage functions. A realistic coverage function was developed for each mode of transportation. The BIP model was compared with two heuristics, Greedy and GRASP. The results showed that GRASP can obtain solutions with performance close to the BIP problem’s solution, in significantly less time. For the largest problem instance Gurobi could solve the BIP problem, the heuristics obtained a solution that performs within 0.18% of BIP problem’s solution in 88% less time. While Gurobi would run into computer memory issues for larger problem instances, GRASP was able to solve them.

The methodology was applied across 29 different municipalities in the Netherlands, encompassing large problem sizes with up to 50000 demand points and between 2183 and 30387 potential installation sites. Results showed that both baseline performance of existing AEDs and relocation potential differ widely. Relative improvements ranged from 1.2% to 175.5%. Moreover, by deploying just 5 to 10 additional AEDs substantial improvements in coverage can already be obtained.

### Heuristics

Although the Greedy algorithm already has given good solutions that may serve as a lower bound, GRASP and the BIP formulation led to solutions that were substantially better, justifying the additional complexity. On both the training and evaluation set, the performance of the BIP formulation’s and GRASP’s solution was similar and in practice indistinguishable. Despite GRASP not guaranteeing (near-)optimal solutions, it consistently achieved results very close to optimal across the tested instances.

GRASP requires specification of the parameter $\alpha$. Cycling through a set of $\alpha$ values is one approach; another is using reactive GRASP, which adapts $\alpha$ based on past performance. However, since the number of iterations may be low for large problem instances, we opted not to use reactive GRASP.

The performance of GRASP is influenced by several factors. The computation time of the local search procedure (steepest ascent) scales with the number of AEDs. Truncated coverage functions can improve GRASP’s performance, as fewer values need to be updated following a swap during the local search.

### Distance metric & mode of transportation

Our chosen distance measure is an approximation, which may have a large error compared to the actual distance. Measuring actual distances for each mode of transportation ourselves is infeasible, and using distance approximations from services like Google maps would be expensive. However, even after multiplying Euclidean distances, the absolute error compared to the actual distance remained large. It may thus be worthwhile to invest in better distance approximations or to seek other GIS solutions to obtain more realistic results. Our methodology can accommodate either of these methods.

Although still an approximation, Euclidean distance multipliers performed better than the Minkowski distance $(\sum _{i=1}^n \vert x_i - y_i \vert )^{1/p}$ for any $p\ge 1$ with regard to errors. However, the distance multipliers are context-specific. For instance, our case study demonstrated cycling as the most effective mode of transport for volunteers in the Netherlands, which may not be the case in countries with less developed bicycle infrastructure. The Dutch VRS HartslagNu recommends retrieving an AED either on foot or by bike.

In practice, choice of mode of transportation may depend on total distance and on the location of the OHCA/AED/volunteer. With a small data set of volunteers, we investigated the relationship between distance and chosen mode of transportation, but results remained largely inconclusive.

We note that our model is general and could incorporate the likelihood of different transportation modes based on the location of the OHCA and the AED candidate location. To illustrate, we could use $c_{ij}=\sum _{t \in T}w^t_{ij}f^t(d_{ij})$, where $w^t_{ij}$ represents the probability of choosing mode of transportation, depending on the location of OHCA *i* and an AED at location *j*. To calculate $w_{tij}$, one could use a multinomial logit model, a type of discrete choice model used to predict selections from among discrete alternatives [69]. In our application, the choice set would be {walking, cycling, driving}. A simple model could try to infer the relationship between the transportation choice and the distance between OHCA *i* and AED location *j*. Revealed preferences from volunteers could be obtained from questionnaire and location data.

### Coverage function

Previous studies used 100m or 176.25m binary coverage [22–24, 41, 49], or used an exponential coverage decay function [20]. These studies only considered unguided bystanders, who are often unsuccessful in defibrillation [14] due to the unavailability of nearby AEDs. Our model enhances realism by using a coverage function based on the timeline of events in a volunteer response, leveraging the fact that volunteers have received basic life support training and are informed about both the AEDs’ and the cardiac arrest’s locations, enabling them to cover greater distances.

While the the coverage decay function’s shape can take different forms, our sensitivity analysis revealed that a linear function provides solutions that also perform well on other function shapes. We based our choice of linear shape on the shape of survival functions in the time interval that volunteers will likely arrive with an AED. Otherwise, the sigmoid function emerges as the most robust option, closely followed by the linear function.

Interpreting coverage values can be challenging due to the dependency on the chosen function. An exponential function typically results in low average coverage, making it difficult to achieve perceived high coverage levels. Establishing a coverage target or standard thus becomes a complex task. Decisions on the number of AEDs to deploy can be guided by evaluating their marginal benefit (Fig. 8) or by examining the distribution of distances to the nearest AED (Fig. 6).

### Application to practice

In practice, optimizing AED locations presents substantial challenges, particularly in the absence of a central authority or decision maker regarding AED management and funding. In the Netherlands, HartslagNu partners, which are volunteer-run local foundations, play a crucial role. These partners not only offer resuscitation training but also work to raise public awareness and raise funds to purchase AEDs. Our model and heuristics could assist these organizations in assessing the quality of existing AED placements and identifying locations for new AEDs.

Relocating AEDs is often impractical due to private ownership. Nonetheless, it seems financially worthwhile to identify *which* AEDs could be relocated, if there are any at all. Additionally, since AEDs are generally not moved once placed at a location, it would be more efficient to deploy AEDs in larger batches. If placed only one at a time, the Greedy algorithm gives the optimal location.

The locations chosen to deploy AEDs from the candidate locations may be infeasible in reality. However, minor adjustments to nearby feasible locations are generally acceptable. An alternative strategy is to compile a list of all public and residential buildings, as done by Tierney et al. [22], and align our candidate locations with the nearest viable building. Regardless of the method, ensuring the visibility and accessibility of AEDs, including proper signage [70], is crucial and takes precedence over strict adherence to the exact location suggested by the model.

Annual coverage may fluctuate due to stochastic locations of the OHCA (Table 4). This has implications for the evaluation of the impact of AED locations. To obtain reliable measurements, several years of data may be needed.

### Limitations

We modeled cardiac arrest risk using Kernel Density Estimation (KDE) to approximate its unknown spatial probability distribution. Supported by existing literature, we assumed that this risk remains stable over both space and time.

Our analysis included only AEDs registered in the HartslagNu database. While more AEDs, especially on-site units, likely exist, their typically low usage rates suggest that excluding them does not significantly impact the effectiveness of optimizing additional AED locations. This implies that actual AED coverage may be more extensive than represented in our study.

The temporal availability of AEDs was not factored into our model due to the lack of reliable data. AEDs within shops, buildings, or private properties may be inaccessible after or before a certain time. We assume any additional AEDs will be placed in outdoor cabinets to provide 24/7 accessibility.

### Future directions

The locations models can be expanded by incorporating the location and behavior of volunteer responders in the response to an emergency. The extended models can identify areas lacking sufficient volunteer coverage, especially in regions with high cardiac arrest risk, and help quantify potential improvements. Data on volunteer responses from a VRS can be used to predict response rates and modes of transportation. Furthermore, the duration of activities in the timeline of a volunteer’s response could be modelled as random variables. Moreover, optimizing for health outcomes like quality-adjusted life years would be preferred over optimizing coverage.

## Conclusion

This study proposes an MCLP model for optimizing AED locations, incorporating volunteer responders, various transportation modes, and multiple decaying coverage functions. Real data from 29 municipalities in the Netherlands are used to demonstrate the effectiveness of the proposed method. Results shows that existing AED locations are suboptimal, and strategically placing a small of number of additional AEDs can substantially improve coverage. Strategic placement of AEDs will both reduce the time to AED connection and increase the number of emergencies that have at least one AED in range. With the results of this research, we hope to increase the overall effectiveness of VRS.

## Appendix A: Validation of KDE

This appendix describes an additional experiment to provide validation for the application of KDE in AED optimization. In repeated 10-fold cross-validation (CV), the training data was used to find an AED solution and the unseen data fold was used to evaluate the respective solution. Then, two approaches were compared:

1. Historic data approach, where the training data was used as direct input to the optimization model
2. KDE approach, where KDE was performed on the training data and then a large sample from the KDE was taken as input to the optimization model (i.e. the approach in this paper)

We aimed to compare both approaches fairly and therefore used the BIP formulation instead of the heuristics to guarantee optimal solutions. Let *R* denote the number of repetitions of *K*-fold cross-validation, *I* denote the set of all OHCAs and $V_{rk}\subset I$ denote the subset of OHCAs in test fold $k \in [K ]$ of repetition $r \in [R ]$. Let $Y_{rk}$ be the vector of AED locations obtained from the solution of the BIP without any knowledge of OHCAs $V_{rk}$. OHCAs $I \setminus V_{rk}$ were used as input to the BIP directly or used to perform KDE on. The average test set coverage is then defined as $\zeta _{rk} = \frac{100\%}{\mid V_{rk} \mid } \sum _{i \in V_{rk}} \max _{j \in Y_{rk}} c_{ij}$, where $c_{ij}$ are the coverage values as defined in Sections 3 and 4. The overall outcome of this experiment is calculated as the average coverage across all test folds: $\zeta = \frac{1}{RK}\sum _{r \in \mid R \mid }\sum _{k \in \mid K \mid } \zeta _{rk}$.

In this experiment, 50 repetitions of 10-fold CV were performed and a grid of 200m between candidate locations was used. 20,000 OHCAs locations were sampled each time for the KDE approach. Lastly, a permutation test was used to test whether the difference in performance of the two approaches was statistically significant.

While we presented 29 real-life problem instances in this study, conducting KDE validation for all instances was computationally infeasible. Therefore, we selected a representative sample of five municipalities.

**Table 8:** Comparison of using historic data and KDE as input to AED optimization in cross-validation

Municipality	Mean coverage $\zeta$ on test folds: Historic data approach	Mean coverage $\zeta$ on test folds: KDE approach	Mean difference (KDE - historic)	p-value
Zaanstad	42.08%	42.50%	0.42%	0.036
Den Helder	55.32%	57.01%	1.69%	p<0.001
Hollands Kroon	43.10%	45.40%	2.30%	p<0.001
Texel	46.44%	45.73%	-0.71%	0.193
Drechterland	43.67%	44.09%	0.42%	0.591

Table 8 shows that for Zaanstad, Den Helder, and Hollands Kroon the KDE approach led to substantially higher coverage than the historic data approach and we reject the null hypothesis that the mean difference is equal to 0 at a significance level of 0.05. While for Texel and Drechterland the KDE approach seems to perform worse and better than the historic data approach, respectively, there is no evidence that the two approaches are not equal (p-values 0.193 and 0.591, respectively).

## Footnotes

1 Synonyms are citizen responder, lay responder, or lay rescuer
